# Effects of Ionic Liquids on the Cylindrical Self-Assemblies Formed by Poly(ethylene oxide)–Poly(propylene oxide)–Poly(ethylene oxide) Block Copolymers in Water

**DOI:** 10.3390/polym16030349

**Published:** 2024-01-28

**Authors:** Aikaterini Tsoutsoura, Zhiqi He, Paschalis Alexandridis

**Affiliations:** Department of Chemical and Biological Engineering, University at Buffalo, The State University of New York (SUNY), Buffalo, NY 14260-4200, USAzhiqihe@buffalo.edu (Z.H.)

**Keywords:** polyethylene glycol, Poloxamer, Pluronic, surfactant, micelle, polymer electrolyte

## Abstract

Aiming at the fundamental understanding of solvent effects in amphiphilic polymer systems, we considered poly(ethylene oxide)–poly(propylene oxide)–poly(ethylene oxide) (PEO-PPO-PEO) block copolymers in water mixed with an ionic liquid—ethylammonium nitrate (EAN), 1-butyl-3-methylimidazolium hexafluorophosphate (BMIMPF_6_), or 1-butyl-3-methylimidazolium tetrafluoroborate (BMIMBF_4_)—and we investigated the hexagonal lyotropic liquid crystal structures by means of small-angle X-ray scattering (SAXS). At 50% polymer, the hexagonal structure (cylinders of self-assembled block copolymer) was maintained across the solvent mixing ratio. The effects of the ionic liquids were reflected in the characteristic length scales of the hexagonal structure and were interpreted in terms of the location of the ionic liquid in the self-assembled block copolymer domains. The protic ionic liquid EAN was evenly distributed within the aqueous domains and showed no affinity for the interface, whereas BMIMPF_6_ preferred to swell PEO and was located at the interface so as to reduce contact with water. BMIMBF_4_ was also interfacially active, but to a lesser extent.

## 1. Introduction

The structural polymorphism of block copolymers is greatly enhanced by the addition of solvents that interact selectively with different polymer blocks [1,2]. For example, at a certain block composition, the self-assembled structure is fixed in the absence of a solvent, but it can be modified in the presence of a selective solvent due to the swelling of one type of block relative to the other [3,4]. Ternary systems of poly(ethylene oxide)–poly(propylene oxide)–poly(ethylene oxide) (PEO-PPO-PEO) block copolymers, a selective solvent for PEO (water), and a selective solvent for PPO (e.g., p-xylene, n-butyl acetate, or butan-1-ol) form a variety of thermodynamically stable lyotropic liquid crystal (LLC) phases, including water-continuous, oil-continuous (reverse or water-in-oil), and bicontinuous structures of self-assembled block copolymers [1,5]. The effects of glycols (e.g., glycerol, propylene glycol, ethanol), which are intermediate in polarity between water and oil, in the structure and stability regions of PEO-PPO block copolymer LLCs have been discussed [6].

Among solvents that support amphiphilic self-assembly, ionic liquids (ILs) attract considerable attention [7,8,9,10,11]. Ionic liquids are organic salts that are molten at ambient temperature, consisting of an asymmetric organic cation and an inorganic anion [12,13]. Ionic liquids possess unique physicochemical properties due to their liquid interionic structure and dynamics [14,15], including low volatility, high density, relatively low viscosity, low flammability, high ion conductivity [16], a large electrochemical window, and electrochemical and thermal stability [17]. Ionic liquids are being investigated in several areas of polymer science for challenging applications [18,19,20,21,22]. More specifically, combinations of polymers and ionic liquids may be employed as polymer electrolytes in fuel cells and in solid-state lithium batteries [23,24,25]. 

Ionic liquids that are selective for PEO support the self-assembly of PEO-PPO-PEO block copolymers [4]. Water is a selective solvent for the PEO block. The introduction of water to a neat ionic liquid weakens the electronic density between anions and cations [26], and hydrogen bonds of different strength are formed between the water and anions, depending on the anion [27,28,29]. It is of high interest to understand how block copolymer self-assemblies respond to the presence of both ionic liquids and water, especially given the specific interactions within the solvent mixture. 

Relatively few studies have investigated the structuring of amphiphiles in binary mixtures of water or molecular solvents with ionic liquids. In prior research, we studied the micelles of a relatively hydrophobic PEO-PPO-PEO block copolymer, Pluronic P123 (EO_20_PO_70_EO_20_), formed in aqueous solutions containing ionic liquids [8,9]. We found that the protic ionic liquid EAN promotes micellization, whereas the aprotic ionic liquid BMIMBF_4_ hinders micellization [8]. Small-angle neutron scattering (SANS) analysis of the micelle structure revealed opposite effects of EAN compared to BMIMBF_4_: an increase in the micelle core radius and association number (i.e., the average number of block copolymer molecules per micelle), and a decrease in the micelle shell thickness, indicating dehydration, upon the addition of EAN; a decrease in the micelle core radius and association number, and an increase in the shell thickness, indicating improved solvation, upon the addition of BMIMBF_4_ [9]. BMIMBF_4_ can interact with the alkyl groups of PPO segments through van der Waals interactions, and it forms hydrogen bonds with PEO segments, both of which assist the PEO-PPO-PEO solvation. Thus, the dehydration of PEO-PPO-PEO molecules caused by the aprotic ionic liquid BMIMBF_4_ is not as significant as that caused by the protic ionic liquid EAN.

The surfactant Laureth-4 (C_12_EO_4_), or Brij30, forms a lamellar LLC structure in mixtures of water and BMIMPF_6_. It was suggested that BMIMPF_6_ localized within the polar domain of the lamellar structure [30]. The nonionic surfactant Brij97 in binary water–BMIMPF_6_ and water–BMIMBF_4_ mixtures forms both lamellar (planar) and hexagonal (cylindrical) structures. While interactions between the ionic liquids and the hydrocarbon tails of the surfactant cannot be excluded, the aforementioned ILs were suggested to partition in the polar domain (consisting of water and the hydrophilic surfactant moiety) of the self-assemblies [31]. Specifically, BMIMBF_4_ was suggested to be located in the water layer of the polar domain, while BMIMPF_6_ was claimed to penetrate the EO segments of the surfactant, resulting in an increased interfacial area per surfactant molecule, as evidenced by SAXS [31]. Ternary systems of oleyl polyoxyethylene (20) ether (C18:1E20), water, and ionic liquids (BMIMBF_4_ or BMIMPF_6_) formed cubic structures assigned to the crystallographic group lm3m that were stable across different water dilution contents. The cubic structures formed in the mixture with BMIMPF_6_ exhibited higher lattice spacing values at the very same water content. This was attributed to the different location of the ionic liquids in the microstructure: BMIMBF_4_ was considered to be located in the water layer of the cubic structure, whereas BMIMPF_6_ was considered to be located among the PEO chains [32]. Drummond and coworkers [33] reported that the addition of water had a very small effect on the self-assembly of Pluronic P123 in EAN, and that EAN had no interaction with the PPO domains of the block copolymer, and its main interaction was with water.

The molecular interactions between amphiphiles and ionic liquids, as reflected in the structural characteristics of the amphiphilic self-assemblies, are the subject of an ongoing investigation in our group. We recently reported the phase behavior and structure of PEO-PPO-PEO block copolymers in binary mixtures with ionic liquids of different characteristics [4]. The hypothesis behind this study is that, when self-assembly takes place in a mixture of solvents, each solvent may participate in the self-assembly in different ways depending on its relative interaction with the amphiphile. Herein, we discuss the synergistic effects of representative ionic liquid solvents with the most common molecular solvent, i.e., water, as self-assembly-supporting media. We selected a PEO-PPO-PEO block copolymer (Pluronic P105) that consists of 50% PEO and 50% PPO, and we investigated the effects of three different ionic liquids on the block copolymer’s hexagonal LLC structures (i.e., ordered cylindrical assemblies) formed in water. The values of the characteristic length scales were obtained for all ternary systems and were correlated with the location of the ionic liquid in the domains formed by self-assembly.

## 2. Materials and Methods

### 2.1. Materials

**Figure 1:**
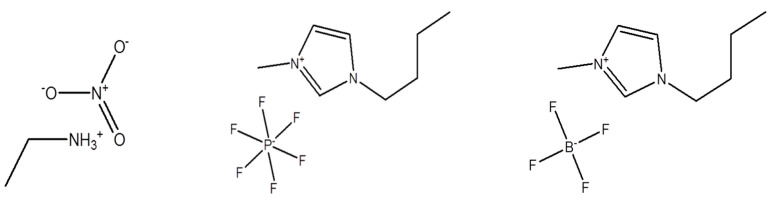
Molecular structures of ethylammonium nitrate (EAN), 1-butyl-3-methylimidazolium hexafluorophosphate (BMIMPF_6_), and 1-butyl-3-methylimidazolium tetrafluoroborate (BMIMBF_4_).

The Pluronic P105 poly(ethylene oxide)–poly(propylene oxide)–poly(ethylene oxide) block copolymer was obtained as a gift from BASF Corp. and used as received. On the basis of its nominal molecular weight of 6500 and 50% PEO, Pluronic P105 consists of 58 PO segments and (2 × 37) EO segments and can be represented as EO_37_PO_58_EO_37_. Ethylammonium nitrate (EAN) (CH_3_CH_2_NH_3_^+^NO_3_^−^) was purchased from IoLiTec Ionic Liquids Technologies GmbH (Heilbronn, Germany). 1-Butyl-3-methylimidazolium hexafluorophosphate (BMIMPF_6_) and 1-butyl-3-methylimidazolium tetrafluoroborate (BMIMBF_4_) were purchased from Sigma-Aldrich, St. Louis, MI, USA. The ionic liquids’ chemical structures are shown in Figure 1. We selected these specific ionic liquids for study for a number of reasons: all three are known to be good solvents for PEO, thus providing the selectivity required for promoting the self-assembly of PEO-PPO block copolymers; they cover both the aprotic and the protic classes, and they have been among the best studied in terms of both fundamentals and applications. The ionic liquids were stored in a desiccator to avoid exposure to atmospheric humidity. Millipore purified water was used for all samples.

The samples were prepared individually at a constant block copolymer concentration of ~60 wt.%, and the appropriate amounts of first water and then ionic liquid were added. The samples were prepared in glass tubes that were flame-sealed upon the addition of the ionic liquid. Subsequently, each sample was centrifuged repeatedly in both directions over the course of several days to facilitate macroscopic mixing. Thereafter, the samples were kept at room temperature and were checked periodically for possible phase separation. The texture of the one-phase homogeneous samples was examined by means of polarized light. All samples discussed herein were transparent, an indication of their homogeneity at the sub-micrometer scale.

### 2.2. Small-Angle X-ray Scattering (SAXS)

SAXS was used to study the self-assembled nanostructures. The experiments were performed at 25 °C using a Nano-STAR instrument (Bruker-AXS, Madison, WI, USA) operating at 40 kV and 35 mA. The sample-to-detector distance was 1015 mm. The X-ray wavelength used was 0.1542 nm (Cu Kα). The angular distribution of the scattered electrons was recorded using a two-dimensional detector [34]. All samples examined by SAXS were single-phase and had been equilibrated for at least one month before the tests. The scattering intensity was derived after averaging the intensity of all points in the 2D detector space for a scattering vector value, *q*, defined as follows:

(1) $$q=\frac{4\pi }{\lambda }sin\left(\frac{\theta }{2}\right)$$

where *θ* is the angle between the incident beam and the scattered radiation.

**Figure 2:**
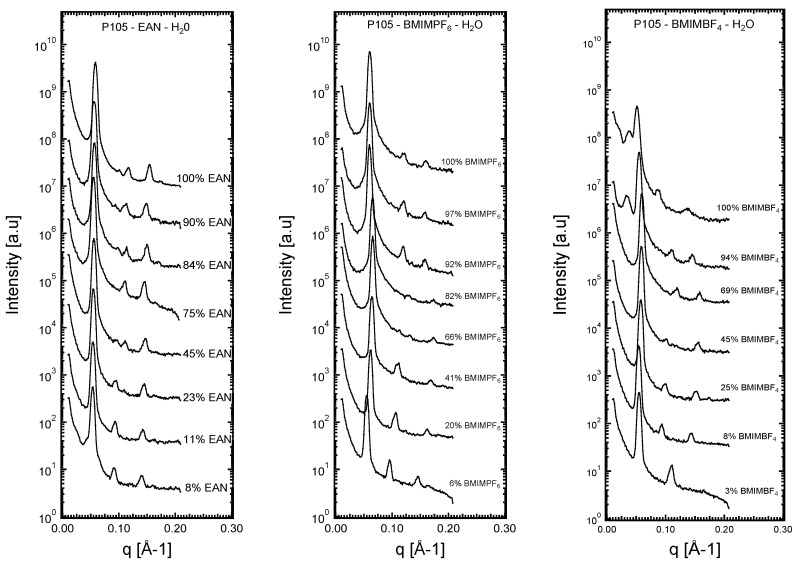
SAXS diffraction patterns from representative samples in the hexagonal lyotropic liquid crystalline regions of the EO_37_PO_58_EO_37_–ionic liquid–water isothermal (25 °C) ternary systems. Compositions are presented in Table 1, Table 2 and Table 3.

The characteristic length scales of the obtained liquid crystalline phases were established through analysis of the SAXS diffraction patterns. SAXS profiles (i.e., *q* wave vectors with respect to intensity) are plotted in Figure 2 for each of the ternary systems (Pluronic P105–ionic liquid–water).

The obtained Bragg diffraction peaks were relatively sharp, as shown in Figure 2, facilitating the assignment to q and structure. The structural analysis using the SAXS data was carried out following a procedure discussed previously [5]. The relative position of the Bragg peaks was initially assessed by examining the patterns and noting the peak positions. Thereafter, the same type of assessment was performed by employing the Igor Multipeak Fitting program (Wavemetrics, Inc., Lake Oswego, OR, USA). The relative positions of the peaks obeyed the relationship 1:3^1/2^:2:7^1/2^, which is characteristic of a hexagonal structure [3,5]. The lattice spacing α for hexagonal structures represents the distance between the centers of adjacent cylinders, can be directly obtained from the SAXS diffraction patterns (given by the position, q*, of the first and most intense diffraction peak), and is defined as follows:

(2) $$a=\frac{4\pi }{q^{*}\sqrt{3}}$$

To characterize the solvophobic domain of the hexagonal microstructure, we defined the radius of the cylinders as follows:

(3) R=a32πf12

In order to define *R*, we assumed that there was a sharp interface between the solvophilic and solvophobic microdomains (this assumption also holds for interfaces that are not sharp, as can be the case in block copolymers, as long as the distributions on either side of the interface balance out) and that *f* represents the total volume fraction of the solvophobic components at the specific composition of the ternary system. 

The distance between the solvophobic cylinders (or, in other words, the thickness of the water–ionic liquid solvent mixture) is given by

(4) $$d_{W+IL}=a-2R$$

Through simple geometric relations, and aiming at gaining insight on the packing of the block copolymers, we defined the interfacial area per PEO block (α*_p_*) [3,5], or the average area that a PEO block of the PEO-PPO-PEO block copolymer occupies at the interface between the solvophilic and solvophobic domains, as follows:

(5)  ap=vpaΦint2π3f12

where Φ*_int_* is the volume fraction of all of the components contributing to the solvophilic–solvophobic interface, and *ν_p_* is the volume of one block copolymer molecule.

Table 1, Table 2 and Table 3 report the compositions of each component of each of the three ternary systems, along with the structural parameters obtained from SAXS. The experimental uncertainty in determining q* is ±1%, and this propagates to the structural parameters obtained using q*.

### 2.3. Interfacial and Solvophobic Volume Fractions

In order to obtain a quantitative insight into the structural characteristics of the self-assemblies, we introduced expressions for the solvophobic and interfacial volume fractions. Initially, we assumed strong segregation of the polymer blocks. Therefore, the solvophobic domain consisted only of the PPO blocks, resulting in the solvophobic volume fraction being equal to the volume fraction of the PPO block of the PEO-PPO block copolymer. We took the polar microdomain to comprise the PEO blocks, water, and all of the ionic liquid. Under these considerations, we defined the solvophobic volume fraction as follows:

(6) $$f=0.54\Phi_{p}$$

where Φ*_p_* is the volume fraction of the block copolymer in the ternary system, and 0.54 is the volume fraction of the PPO block in the P105 macromolecule (the PPO weight fraction is 0.50, which makes up a 0.54 volume fraction). The volume of one P105 macromolecule is $\nu_{p}\approx 10300\;{Å}^{3}$. If we assume that the block copolymer is the only surface-active component in all of the ternary systems, then the interfacial volume fraction would be equal to the polymer volume fraction:

(7) $$\Phi_{int}=\Phi_{p}$$

The values of the characteristic length scales obtained after the implementation of these assumptions, along with the respective trends, are reported and discussed in the Section 3. 

No contribution of the ionic liquid to the solvophobic volume fraction was accounted for above. Even though interactions between the ionic liquid and PPO cannot be completely excluded, the interactions between the ionic liquid (and its polar nanodomains) and PEO, as well as water, were the dominant ones. We noted that BMIMBF_4_ and BMIMPF_6_ were completely immiscible with oils such as hexane or decane, and vice versa [35]. Furthermore, PPO is soluble in EAN only up to 1 wt.%, with a cloud point around 34 °C [36].

Next, we need to consider a second set of assumptions in defining the interfacial volume. We still suggest that the solvophobic volume fraction consists of PPO ($f=0.54\Phi_{p}$) without any ionic liquid partitioning. Thus, the estimation of the radius of the cylinders of the solvophobic domains is not affected, because the radius depends only on the lattice parameter and the total solvophobic volume fraction. Consequently, there is no effect on the estimation of the thickness of the solvent mixture layer. 

For the interfacial volume, however, we suggest that the block copolymer is not the only surface-active component in the ternary systems. The ionic liquids, due to their inherent amphiphilic nature, have the potential to exhibit surface-active behavior; therefore, we can redefine the interfacial volume fraction as follows:

(8) $$\Phi_{int}=\Phi_{p}+\chi \Phi_{IL}$$

where *χ* is the fraction of the ionic liquid that participates (i.e., is located) in the interface.

In the Section 3, both assumptions for the interfacial volume are taken into consideration, and their effects on the characteristic length scales are discussed so as to validate the most appropriate definitions.

## 3. Results and Discussion

### 3.1. Effects of Ionic Liquids on Hexagonal Structures at Constant Block Copolymer Contents

**Figure 3:**
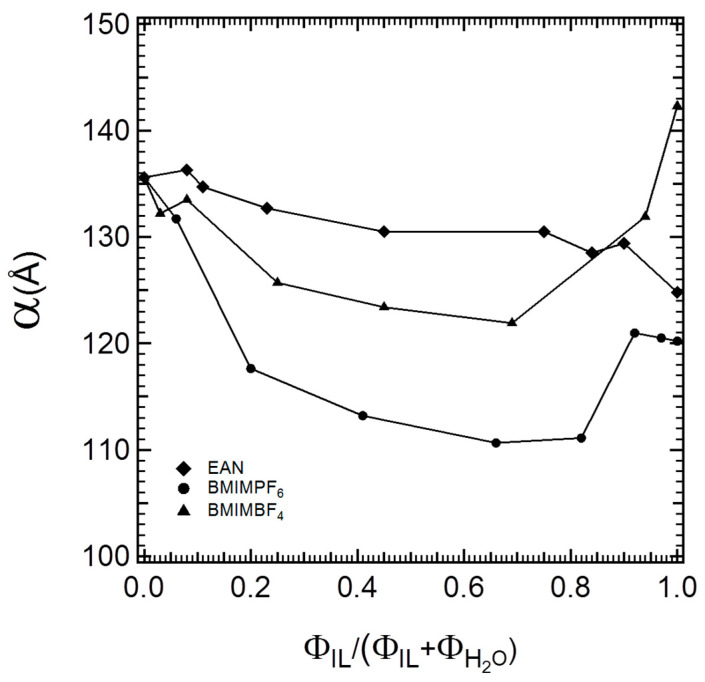
Lattice spacing plotted as a function of the ionic liquid volume fraction relative to the volume fraction of the ionic liquid + water solvent.

The SAXS patterns in Figure 2 show that hexagonal structures formed in all ternary isothermal systems, regardless of the type of the ionic liquid and its miscibility with water. Higher numbers of peaks and/or more intense peaks were recorded with EAN. The two aprotic ionic liquids, particularly BMIMPF_6_, showed a tendency to decrease the intensities of high-order peaks. In all three systems, the hexagonal structures were located between the micellar cubic and the lamellar regions, indicating that their morphology should be water-continuous. In Figure 3, the lattice spacing is plotted as a function of the ionic liquid volume fraction relative to the volume fraction of the solvent mixture. 

The lattice spacing of the hexagonal structures in each ternary isothermal system tended to decrease with the addition of increasing amounts of ionic liquid relative to water (i.e., the content of the combined solvent remained fixed). Alternatively, the gradual replacement of water portions from the solvent mixture with the respective ionic liquid decreased the lattice parameter because the interfacial area increased. PEO was still the only block that was preferentially solvated. However, the ionic liquids were not as “bad” as solvents for PPO as water was: the solvophobic interactions between the ionic liquids and PPO were weaker than that between water and PPO. The lattice spacings in EAN–water mixtures were similar to the value in plain water, and their decrease with EAN content was close to linear. The behavior of the imidazolium ionic liquids was different. The lattice spacings decreased with IL content up to about 65% IL in its mixture with water, with BMIMPF_6_ being more effective in decreasing the lattice parameter. At higher IL contents, the lattice spacings in the aqueous BMIMPF_6_ and BMIMBF_4_ systems increased. The IL–water ratio where the “minimum” lattice spacing occurred was consistent with the trends observed in other properties in such water–IL mixtures. The changes in the lattice parameter reflect the balance of intermolecular forces between the water, ionic liquid, and PEO-PPO block copolymer, as discussed next.

### 3.2. Location of Ionic Liquids in the Self-Assembled Block Copolymer Microstructure

The differences observed in the lattice spacing between the three ionic liquids could be related to changes in the solvophobic and/or interfacial volumes in the ternary systems. As discussed in the Section 2.3, we considered the solvophobic volume fraction to be constant in all three systems and equal to the volume fraction of the solvophobic PPO block. Thus, the variation in the lattice parameter is the result of the different contribution of each ionic liquid to the interfacial volume fraction. The ionic liquids displayed different selectivities while interacting with Pluronic P105 in binary systems (i.e., no water present) [4], as well as different interactions with water [8]. Both of these phenomena significantly influence the way in which each ionic liquid localizes in the block copolymer domains of the hexagonal structure, and they are reflected in the obtained values of the lattice parameters.

**Figure 4:**
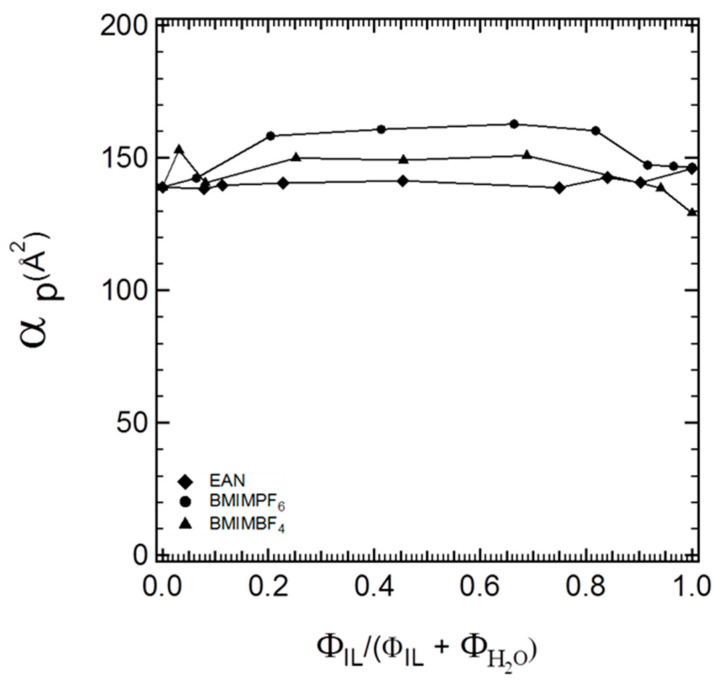
Interfacial area per PEO block plotted as a function of the ionic liquid volume fraction relative to the volume fraction of the ionic liquid + water solvent.

More specifically, in the three-component (P105–IL–water) hexagonal microstructures, the values of the lattice parameters followed the order α_P105-W-EAN_ > α_P105-W-BMIMBF4_ > α_P105-W-BMIMPF6_. Conversely, in the hexagonal structures of binary block copolymer and ionic liquid systems, the lattice parameters were in the following order: α_P105-BMIMBF4_ > α_P105-EAN_ > α_P105-BMIMPF6_ [4]. The difference in the lattice spacing rankings for binary and ternary systems suggests that each ionic liquid participates at the interface (interfacial volume fraction) in different ways, depending on the specific interactions of each ionic liquid with water. Part of the ionic liquid interacts preferentially with water, while part of it is expected to favor the interface. The interfacial area per PEO block is plotted in Figure 4 as a function of the ionic liquid volume fraction relative to the volume fraction of the ionic liquid + water mixed solvent.

**Figure 5:**
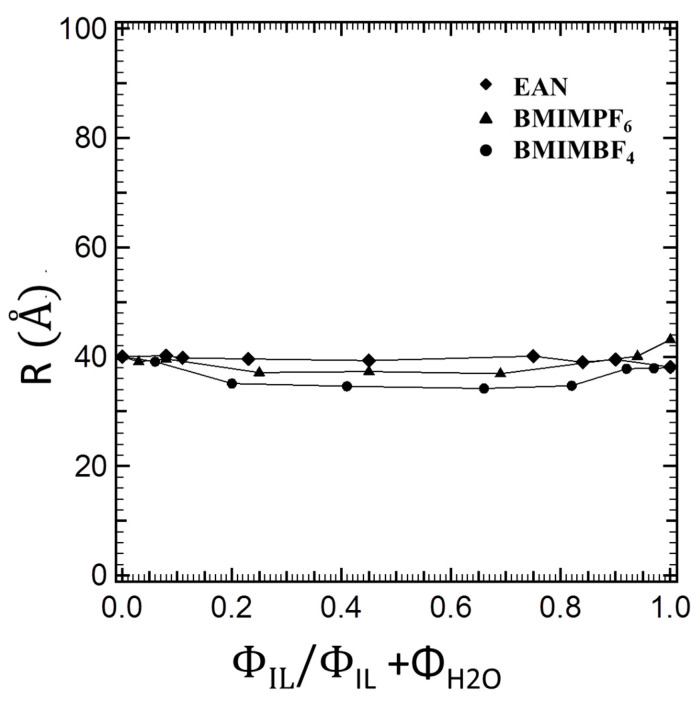
Radii of the solvophobic domains of the block copolymer cylinders plotted as a function of the ionic liquid volume fraction relative to the volume fraction of the ionic liquid + water solvent.

In Figure 4, we can observe different polymer interfacial areas for the three ionic liquids at the same block copolymer composition. While the interfacial area per block copolymer molecule should be independent of the structure where it is located, this is not the case in Figure 4. For all three ionic liquids, we obtained increasing values for the interfacial areas by increasing the ionic liquid content, which implies that the initial assignment of the interfacial volume fraction was not accurate and, hence, that the ionic liquid partitions at the solvophilic/solvophobic interface by contributing to the swelling of the PEO blocks. Therefore, we need to employ additional assumptions (as discussed in the Section 2.3) and calculate the contribution of the ionic liquid to the interfacial volume. Moreover, by accounting for no ionic liquid partition in the solvophobic volume fraction, the radius of the solvophobic domains of the block copolymer cylinders depends solely on the composition of the block copolymer, i.e., on the total composition of the PPO block at the very specific composition of the ternary system. As shown in Figure 5, the solvophobic cylinders were of almost invariant thickness. 

**Figure 6:**
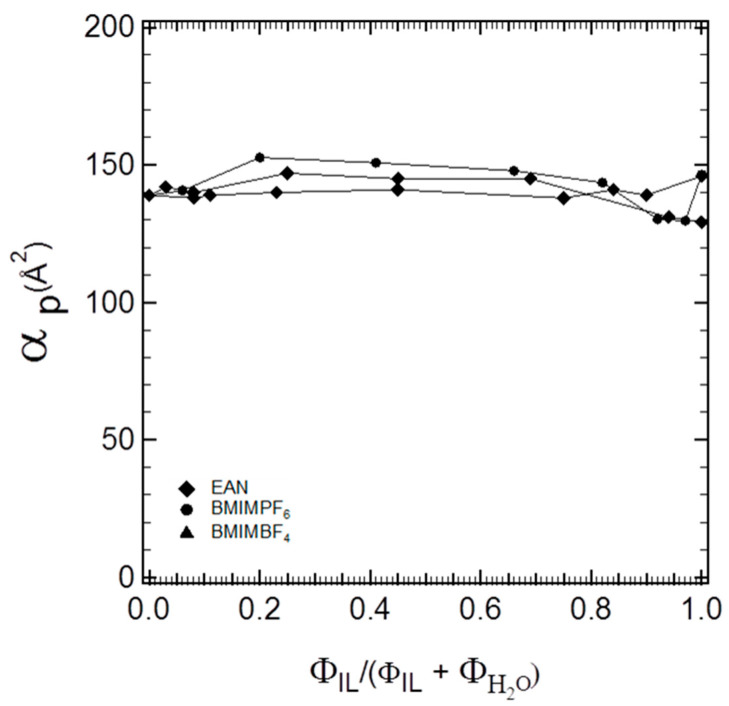
Interfacial area per PEO block plotted as a function of the ionic liquid volume fraction relative to the volume fraction of the ionic liquid + water solvent with partitioning of the ionic liquid in the interfacial volume fraction.

Aiming for a quantitative assessment of the ionic liquid partitioning at the interface, we employed Equation (6) and deduced the fraction of each ionic liquid participating in the stabilization of the interface. We achieved this by maintaining the interfacial area values in all of the systems at the same invariant block copolymer concentration, as illustrated in Figure 6. The “interfacial area invariance” criterion suggests that the area per PEO block in such copolymers depends on the ratio of polar (PEO block) to less polar (PPO block) domains and is expected to be independent of the added solvent at the very same block copolymer composition, provided that the correct assumptions for the less polar and interfacial volumes have been established. This criterion has been established as a powerful method to provide insight into the location of the solvent in the microstructure, as has been observed in ternary systems containing diverse contents of PEO, water, and relatively hydrophobic oils or relatively polar glycols [1,6]. Thus, by keeping the interfacial area almost invariant at the same block copolymer composition for the same ionic liquid/water mixture, as well as for diverse ionic liquid/water mixtures, as illustrated in Figure 6, we can deduce the fraction of the ionic liquid that partitions at the interface. These contributions are discussed later in detail for every ternary system. The values for interfacial areas that take into account the partition of the ionic liquid into the interfacial volume fraction are presented in Table 4. To obtain these values, we adjusted *χ* in Equation (8) so that the α*_p_* values obtained from Equation (5) at the same block copolymer concentration were approximately the same.

In the following, we elaborate on the partitioning of the ionic liquid in the interfacial region for each of the ternary systems.

#### 3.2.1. EO_37_PO_58_EO_37—_EAN–Water System 

EAN is the most extensively investigated protic ionic liquid [37,38,39]. Protic ionic liquids (PILs) are formed by the transfer of a proton from a Brønsted acid to a Brønsted base [40]. Structurally, EAN presents hydrogen bond donor and acceptor sites that enable the formation of three-dimensional hydrogen bond network similar to that of water [41]. EAN is miscible with water, and hydrogen bonds are formed between its ions (C_2_H_5_H_3_N^+^ and NO_3_^−^) and water molecules. Even a significant presence of water does not alter the structural order of EAN [42]. EAN is a good solvent for PEO [43], and its aforementioned hydrogen bond network is the one promoting the solvation of this polymer. The PEO blocks of the PEO-PPO-PEO block copolymer are solvated by EAN, leading to segregation from the PPO block. The ethylammonium cation (CH_3_CH_2_NH_3_^+^) is suggested to interact with the oxygen atom of PEO segments, segregating them from PPO, leading finally to microphase separation. PPO has been reported to be soluble in EAN only up to 1 wt.%, with a cloud point around 34 °C [36].

**Figure 7:**
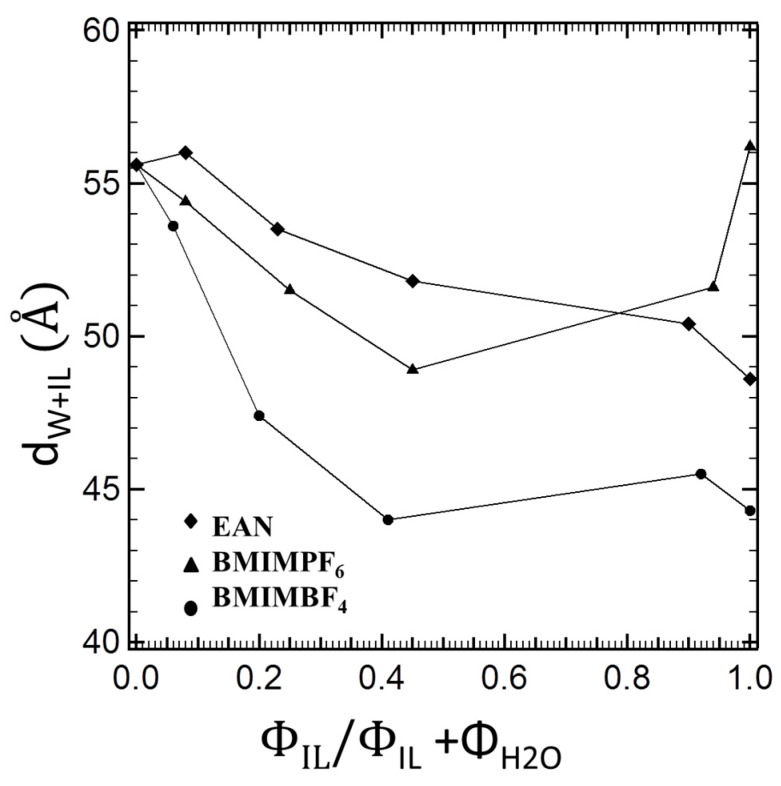
Thickness of the water–ionic liquid layer plotted as a function of the ionic liquid volume fraction relative to the volume fraction of the ionic liquid + water solvent.

The “interfacial area invariance” criterion (refer to discussion around Table 4) suggests that EAN participates by only 2% in the total interfacial volume. Being structurally reminiscent of water, EAN is the least surface-active ionic liquid of those considered here. However, EAN, aside from swelling the PEO block together with water, interacts well with water, maintaining the thickness of the water-continuous region, as shown in Figure 7. On the one hand, starting from the hexagonal structure of the binary P105–water system and replacing the water with an ionic liquid, we insert a solvent with which the PPO has weaker solvophobic interactions into the system, while still swelling the PEO block; hence, the interfacial area is allowed to increase, leading to a smaller lattice parameter than water. On the other hand, starting from the hexagonal structure of the binary P105–EAN system and adding a highly selective solvent such as water, the unfavorable interactions with PPO and the ongoing swelling of PEO lead to relatively decreased values of the interface and higher lattice parameters. From both points of view, EAN, being fully miscible or even interchangeable with water, is located primarily in the water-continuous region, and only in a very small portion in the interface.

#### 3.2.2. EO_37_PO_58_EO_37_–BMIMBF_4_–Water System

BMIMBF_4_ is completely miscible with water at room temperature. In water-rich mixtures, BMIMBF_4_ forms self-assembled micellar structures [44,45,46] at room temperature and shows an upper critical solution temperature of 278 K [44]. According to Fourier-transform infrared and Raman spectroscopy results, when small amounts of water are added to BMIMBF_4_ (weight fraction—X_w_ < 0.1), the water molecules do not cluster, disrupt the hydrogen bonding between the fluorine anions and the polar head of the imidazolium ring (C-H proton and particularly C(2)-H of the imidazolium ring), and individually interact with anions, forming symmetrical hydrogen-bonded complexes (anion…H-O-H…anion) [47]. When increasing the water content (X_w_ = 0.1), the water–water interactions compete with the water–anion interactions, resulting in the formation of water clusters before the complete saturation of all of the anions available for hydrogen bonding with water molecules (X_w_ = 0.70). The water network formation starts at around X_w_ = 0.51 and increases rapidly up to X_w_ = 0.70, after which the network keeps expanding slowly. The water aggregates are embedded into the polar network of imidazolium rings–anions and, when it comes to the micelle formation of BMIMBF_4_ in water, they determine the spatial segregation of the polar heads from the hydrophobic alkyl tails. At X_w_ = 0.70, the water-networked molecules disrupt the interactions between the fluorine anions and the positively charged BMIMBF_4_ heads. At higher water contents, the ring–anion bonds are lost, the structural organization of BMIMBF_4_ is weakened, and the formation of water clusters is decreased [47]. Furthermore, it has been reported that BMIMBF_4_ is a good solvent for PEO. SANS investigation of PEO solutions in BMIMBF_4_ demonstrated that BMIMBF_4_ acts as good solvent for PEO, which organizes itself in random coils [48]. Owing to its chemical nature, part of BMIMBF_4_ contributes to the apolar domain, while part of it acts as a cosurfactant and as a solute in the aqueous phase located at the interface.

By using the “interfacial area invariance” criterion, we established the surface activity of BMIMBF_4_m which participates at the interface by 10% (i.e., 10% of the total interfacial volume). BMIMBF_4_ swells the PEO block more than EAN, as has been established by comparison of the respective binary block copolymer + ionic liquid systems [4]. Being miscible with water, BMIMBF_4_ increases the thickness of the water layer (albeit less than EAN, as it is engaged more in swelling PEO and stabilizing the interface (Figure 6)). By replacing the highly selective water with a less selective ionic liquid, again we obtained decreased lattice parameters due to increased interfacial areas and weaker solvophobic interactions than those of water. When adding water to the system of P105-BMIMBF_4_, it seems that the ionic liquid–water interactions dominate. Less of the BMIMBF_4_ interacts with the block copolymer, and the PEO blocks are steadily swelled; hence, the interfacial areas increase and the lattice parameters decrease.

#### 3.2.3. EO_37_PO_58_EO_37_–BMIMPF_6_–Water System

Possessing a highly hydrophobic anion [PF_6_]^−^, BMIMPF_6_ is not fully miscible with water and has a solubility of 2.0 ± 0.3 wt.% under ambient conditions [49]. Water clusters are formed in the bulk mixture of water and BMIMPF_6_ due to repulsive interactions between water molecules and the hydrophobic [PF_6_]^−^ anions [50]. Ionic liquids based on 1-alkyl-3-methylimidazolium cations such as BMIMPF_6_ are good solvents for PEO [48,51]. Hydrogen bonding interactions may take place between the ether unit and the acid hydrogen on the imidazolium ring. In particular, molecular dynamics simulations of the molecular interactions between BMIMPF_6_ and PEO indicated that the ionic structure of BMIMPF_6_ is disrupted during the solvation of PEO [51]. The oxygen atoms of PEO chains coordinate with the imidazolium cation, leading to a preferred solvation of the imidazolium ring by oxygen atoms of polymeric chains instead of PF_6_^−^ anions. Experimental results showed that the imidazolium ring may act as a hydrogen bond donor, while the terminal hydroxyl groups as well as the ethoxy groups of PEO can act as hydrogen bond acceptors. In parallel, the [PF_6_]^−^ anion may act as an acceptor, and the terminal hydroxyl groups of PEO may act as donors [52].

The “interfacial area invariance” criterion demonstrated that BMIMPF_6_ participates with the highest fraction at the interfacial volume fraction, i.e., ~30%. BMIMPF_6_, as has been discussed, is hydrophobic, as clearly justified by the thinner water layer displayed in Figure 7. Therefore, it penetrates and swells the PEO groups and, once it is inserted into the P105–water binary system, it weakens the solvophobic interactions, and the interfacial area increases while the lattice parameter decreases. When water is inserted into the binary system P105–BMIMPF_6_, the PEO swelling continues in synergy with BMIMPF_6_, which prefers to swell PEO or be located at the interface so as to avoid contact with water. Hence, again, the interfacial area increases and the lattice parameters decrease.

### 3.3. Comparison of Ionic Liquids’ Location with That of Molecular Solvents

In our previous studies, the role of polar molecular solvents (e.g., ethanol, propylene glycol, glycerol, and glucose) has been investigated in ternary phase diagrams of the same PEO-PPO-PEO block copolymer in the presence of water [6]. The location of molecular solvents in the microstructures of the block copolymer was assigned by employing the same methodology as the current work [6], therefore allowing us a direct comparison. In addition, the ionic liquids employed herein resulted in similar phase sequences in the ternary systems to the one observed for ethanol and propylene glycol, with a bicontinuous cubic phase as a common theme, in contrast to the effects of glycerol and glucose. These molecular solvents can be compared to the ionic liquids in terms of polarity. The polarity of the glycols increases as follows: ethanol < propylene glycol < glycerol < glucose [6,52]. The polarity of these molecular solvents is comparable to the polarity of ionic liquids in terms of dielectric constant values, as can be seen in Table 5.

The effects of the ionic liquids on the lattice parameters and interfacial areas revealed herein are analogous to the effects of ethanol and propylene glycol. The addition of ethanol or propylene glycol increases the interfacial area and decreases the lattice parameter [6]. Conversely, for the more polar glycerol, the effect is the opposite. However, at the same organic solvent content, the decrease in the lattice parameters is higher in the case of the molecular solvents; in other words, the ionic liquids at small percentages in the hexagonal microstructure (5 wt.%) result in higher lattice spacing values than the similarly polar molecular solvents. This comparison is made in Table 6.

The ionic liquids swell PEO and participate in the formation of the interface; however, in contrast with the aforementioned glycols, they do not participate in the solvophobic domain. Although the ionic liquids exhibit similar polarity to these glycols, they do not interact with PPO, they swell PEO more, and they interact with water. The role of ionic liquids is more complex and is the result of their inherent structural complexity. Moreover, the ionic liquids studied here are able to support hexagonal structures even in binary systems with P105 [4], whereas the glycols are not documented as being capable of such behavior [6]. It is possible that other ionic liquids of similar polarity to the above glycols are able to support self-assembly and display similar effects. We should note here that we considered in this study binary mixtures of water and ionic liquids. A recent study [56] of ternary mixtures of water, ethylammonium nitrate, and 1-butyl-3-methylimidazolium iodide has paved the way for amphiphile self-assembly studies in such multicomponent solvents [34].

### 3.4. Comparison to Other Nonionic Surfactants in Mixtures of Water and Ionic Liquid

Ternary systems of Brij97 in water and BMIMBF_4_ or BMIMPF_6_ resulted in the formation of hexagonal LLC. Similar to our systems, the lattice parameter values with the addition of BMIMBF_4_ were higher, while the interfacial areas were lower. The authors excluded any interactions with the apolar (alkyl) part of the surfactant and suggested that both ionic liquids were located in the polar domain (i.e., the hydrophilic part of the surfactant and water). More specifically, and in analogy with our conclusions here, they claimed that BMIMBF_4_ (due its miscibility with water) is located in the water layer, while the hydrophobic BMIMPF_6_ swells the PEO parts [31]. The same location assignment and lattice parameter trends were reported for cubic phases of oleyl polyoxyethylene (20) ether (C18:1E20) surfactant in water and each of the aforementioned ionic liquids [32].

## 4. Conclusions

The effects of ionic liquids on the cylindrical self-assemblies (i.e., hexagonal lyotropic liquid crystal structures) of poly(ethylene oxide)–poly(propylene oxide)–poly(ethylene oxide) (PEO-PPO-PEO) block copolymers in water are elucidated in this paper. For the three different ionic liquids considered here, the hexagonal structure was preserved across the whole spectrum of replacement of water with the ionic liquid.

Macroscopically, the roles of the ionic liquids appear to be similar to one another and comparable to those of polar glycols such as ethanol and propylene glycol. The interfacial curvature is not altered due to the presence of ionic liquid, and the hexagonal (cylindrical) structure is retained. Microscopically, the effects of the ionic liquids follow the same trends but are not the same. The ionic liquids decrease the lattice parameter values and increase the interfacial areas in an analogous manner to the low-polarity alcohols (i.e., ethanol and propylene glycol). However, the lattice spacing values for the alcohols are lower than those afforded by the ionic liquids. In the case of the aforementioned glycols, the effects on the lattice parameters can be attributed to the weakening of the block segregation due to the swelling of the PPO block, in parallel with the role of glycols in the formation of the interface. In the case of the ionic liquids, we did not account for any contribution of the ionic solvent in the solvophobic volume fraction, even though some weak interactions cannot be excluded. We accounted for partitioning of the ionic liquids at the interface, hence revealing the location of the ionic liquids in the self-assembled microstructure.

The aprotic and structurally similar ionic liquids BMIMPF_6_ and BMIMBF_4_ are located in the microstructure to different extents, but they result in analogous macroscopic effects on lattice spacing and interfacial areas. BMIMPF_6_ swells PEO and participates more in the formation of the interface (30% of the interfacial volume). Conversely, BMIMBF_4_ interacts well with water and is located primarily in the polar aqueous domain, constituting 10% of the interfacial volume. Being structurally similar to water, EAN supports the hexagonal structure in a binary system with P105, acting as a selective solvent for PEO. In the ternary system, a very small amount of EAN participates at the interface (2% of the interfacial volume), but the interactions with water dominate, and EAN is located in the aqueous domain of the structure. From the data and analysis presented here, it emerges that the more hydrophobic the ionic liquid, the more it contributes to the formation of the interface, provided that is a good solvent for the solvophilic block.

The knowledge presented here contributes to the understanding of block copolymer self-assembly in selective solvents and provides guidance in the design of ionic-liquid-containing complex fluids and soft materials.

## Figures and Tables

**Table 1:** Values for the weight and volume fractions of the block copolymer and solvents in the EO_37_PO_58_EO_37_–EAN–water ternary system samples, and for the structural parameters obtained from SAXS on these samples. Φ_EAN_/(Φ_ΕAΝ_ + Φ_H_2_O_) is the normalized volume fraction of EAN, and the ratio of EAN/H_2_O refers to the molecular ratio of the solvents in their mixture.

wt.%			Φ_P105_	Φ_H_2_O_	Φ_EAN_	Φ_EAN_/(Φ_ΕAΝ_ + Φ_H_2_O_)	f_(PPO)_	Solvent Mixture				Molecular Ratio EAN/H_2_O	a	$a_{p}$ (Å^2^)	R	d_W+IL_
P105	H_2_O	EAN						H_2_O wt.%	EAN wt.%	Φ_H_2_O_	Φ_EAN_					
0.598	0.402	0.000	0.586	0.414	0.000	0.00	0.32	1.00	0.00	1.00	0.00	-	135.6	139.0	40.0	55.6
0.592	0.370	0.038	0.583	0.384	0.033	0.08	0.32	0.91	0.09	0.92	0.08	0.02	136.3	138.4	40.2	56.0
0.594	0.352	0.054	0.587	0.366	0.047	0.11	0.32	0.87	0.13	0.89	0.11	0.03	134.7	139.7	39.8	55.1
0.599	0.295	0.106	0.598	0.310	0.092	0.23	0.32	0.74	0.26	0.77	0.23	0.06	132.7	140.5	39.6	53.5
0.601	0.198	0.201	0.611	0.212	0.177	0.45	0.33	0.50	0.50	0.55	0.45	0.17	130.5	141.4	39.3	51.8
0.610	0.084	0.305	0.633	0.092	0.275	0.75	0.34	0.22	0.78	0.25	0.75	0.60	130.5	138.8	40.1	50.4
0.592	0.055	0.352	0.619	0.061	0.320	0.84	0.33	0.14	0.86	0.16	0.84	1.06	128.5	142.6	39.0	50.4
0.597	0.033	0.371	0.626	0.036	0.337	0.90	0.34	0.08	0.92	0.10	0.90	1.88	129.4	140.7	39.5	50.4
0.592	0.000	0.408	0.626	0.000	0.374	1.00	0.34	0.00	1.00	0.00	1.00		124.8	146.0	38.1	48.6

**Table 2:** Values for the weight and volume fractions for the block copolymer and solvents in the EO_37_PO_58_EO_37_–BMIMPF_6_–water ternary system samples, along with their structural parameters.

wt.%			Φ_P105_	Φ_H_2_O_	Φ_BMIMPF6_	Φ_BMIMPF6_/ (Φ_BMIMPF6_ + Φ_H_2_O_)	f(PPO)	Solvent Mixture				Molecular Ratio BMIMPF_6_/H_2_O	a	$a_{p}$ (Å^2^)	R	d_W+IL_
P105	H_2_O	BMIMPF_6_						H_2_O wt.%	BMIMPF_6_ wt.%	Φ_H_2_O_	Φ _BMIMPF6_					
0.60	0.40	0.00	0.59	0.41	0.00	0.00	0.32	1.00	0.00	1.00	0.00	-	135.6	139.0	40.0	55.6
0.60	0.37	0.04	0.59	0.38	0.03	0.06	0.32	0.91	0.09	0.94	0.06	0.01	131.7	142.4	39.1	53.6
0.59	0.30	0.11	0.60	0.32	0.08	0.20	0.32	0.74	0.26	0.80	0.20	0.02	117.6	158.3	35.1	47.4
0.61	0.20	0.20	0.63	0.22	0.15	0.41	0.34	0.51	0.49	0.59	0.41	0.06	113.2	160.8	34.6	44
0.60	0.11	0.29	0.64	0.12	0.24	0.66	0.35	0.27	0.73	0.34	0.66	0.17	110.7	162.8	34.2	42.3
0.60	0.06	0.34	0.66	0.06	0.28	0.82	0.35	0.14	0.86	0.18	0.82	0.40	111.1	160.3	34.7	41.7
0.60	0.03	0.38	0.66	0.03	0.32	0.92	0.35	0.06	0.94	0.08	0.92	0.96	121	147.3	37.8	45.5
0.60	0.01	0.39	0.66	0.01	0.33	0.97	0.36	0.03	0.97	0.03	0.97	2.45	120.5	146.9	37.9	44.8
0.61	0.00	0.39	0.67	0.00	0.33	1.00	0.36	0.00	1.00	0.00	1.00		120.2	146.5	38.0	44.3

**Table 3:** Values for the weight and volume fractions for the block copolymer and solvents in the EO_37_PO_58_EO_37_–BMIMBF_4_–water ternary samples, along with their structural parameters.

wt.%			Φ_P105_	Φ_H_2_O_	Φ_BMIMBF4_	Φ_BMIMBF4_/ (Φ_BMIMBF4_ + Φ_H_2_O_)	f(PPO)	Solvent Mixture				Molecular Ratio BMIMBF_4_/H_2_O	a	$a_{p}$ (Å^2^)	R	d_W+IL_
P105	H_2_O	BMIM BF_4_						H_2_O wt.%	BMIMBF_4_ wt.%	Φ_H_2_O_	Φ _BMIMBF4_					
0.60	0.40	0.00	0.59	0.41	0.00	0.00	0.32	1.00	0.00	1.00	0.00	-	135.6	139.0	40.0	55.6
0.60	0.39	0.02	0.59	0.38	0.01	0.03	0.32	0.96	0.04	0.97	0.03	0.003	132.2	142.2	36.4	54
0.60	0.36	0.04	0.59	0.32	0.03	0.08	0.32	0.90	0.10	0.92	0.08	0.009	133.5	140.6	39.6	54.4
0.58	0.30	0.12	0.58	0.22	0.11	0.25	0.32	0.71	0.29	0.75	0.25	0.033	125.7	150	37.1	51.5
0.60	0.20	0.20	0.61	0.12	0.18	0.46	0.33	0.50	0.50	0.55	0.45	0.081	123.4	149.2	37.3	48.9
0.59	0.11	0.30	0.61	0.06	0.27	0.69	0.33	0.27	0.73	0.31	0.69	0.213	121.9	150.9	36.9	48.2
0.59	0.02	0.39	0.62	0.03	0.36	0.94	0.34	0.05	0.95	0.06	0.94	1.531	131.9	138.6	40.1	51.6
0.58	0.00	0.42	0.62	0.01	0.39	1.00	0.33	0.00	1.00	0.00	1.00		142.3	129.2	43.1	56.2

**Table 4:** Interfacial area values (almost invariant) in the hexagonal structures while taking into account the ionic liquids’ partitioning into the interfacial volume.

Φ_EAN_/ (Φ_ΕAΝ_ + Φ_H_2_O_)	α_p_ (Å^2^)	Φ_BMIMPF6_/ (Φ_BMIMPF6_ + Φ_H_2_O_)	α_p_ (Å^2^)	Φ_BMIMBF4_/ (Φ_BMIMBF4_ + Φ_H_2_O_)	α_p_ (Å^2^)
0	139	0	139	0	139
0.08	138.3	0.06	140.7	0.03	141.9
0.11	139.5	0.20	152.7	0.08	139.8
0.23	140.1	0.41	150.8	0.25	147.4
0.45	140.5	0.66	147.9	0.45	145.0
0.75	137.6	0.82	143.6	0.69	144.6
0.84	141.1	0.92	130.3	0.94	131.1
0.90	139.2	0.97	129.7	1	129.2
1	146	1	146.5		

**Table 5:** Dielectric constant values for ethanol, glycerol, propylene glycol, and ionic liquids.

Solvent	ε_s_	Reference
Ethanol	24.3	[6]
Glycerol	40.1	[6]
Propylene Glycol	32	[6]
EAN	26	[53]
BMIMPF_6_	14.1	[54,55]
BMIMBF_4_	14.1	[54,55]
Water	78.5	[6]

**Table 6:** Lattice parameters for 5 wt.% contents of solvents in ternary P105–water–cosolvent systems.

Solvent	α (Å)
Ethanol	126
Glycerol	139
Propylene Glycol	129
EAN	135
BMIMPF_6_	132
BMIMBF_4_	134

## Data Availability

Data are available upon reasonable request.
